# Thiol/Disulfide Balance in Patients with Familial Hypercholesterolemia

**DOI:** 10.1155/2018/9042461

**Published:** 2018-06-12

**Authors:** Özgür Şimşek, Ayşe Çarlıoğlu, Murat Alışık, Efe Edem, Cemile Koca Biçer

**Affiliations:** ^1^Internal Medicine Department, Erzurum Regional Education Research Hospital, Atatürk Mahallesi, Çat Yolu Cd., Palandöken, Yakutiye, Erzurum 25070, Turkey; ^2^Biochemistry Department, Ankara Yıldırım Beyazıt University, Çankırı Caddesi, Çiçek Sokak No. 3, Ankara, Turkey; ^3^Cardiology Department, İzmir Tınaztepe Hospital, Ahmet Piriştina Blv. No. 51 Tınaztepe, Buca, İzmir 35400, Turkey

## Abstract

**Objective:**

Herein, we investigated the balance of thiol/disulfide, with the hypothesis that the balance between disulfides and thiols, which are natural antioxidants, might be disrupted in patients with familial hypercholesterolemia, which eventually leads to endothelial damage.

**Methods:**

In this study, we evaluated 51 patients diagnosed with familial hypercholesterolemia and 81 healthy subjects. Blood samples were taken from the patients after a minimum of 12 hours of fasting; samples were immediately centrifuged, stored in Eppendorf tubes, and preserved at −80°C.

**Results:**

This study found that thiol levels are significantly lower in patients with familial hypercholesterolemia, whereas disulfide levels are higher (independent of age, gender, and body mass index). This means that in such patients, the thiol/disulfide balance changes in favour of disulfide.

**Conclusions:**

In this study, we found that the thiol/disulfide balance in patients with familial hypercholesterolemia is disrupted in favour of disulfide.

## 1. Introduction

Currently, cardiovascular disease (CVD) is the most significant cause of mortality, and hyperlipidaemia is a major risk factor for atherosclerosis, which is the main pathogenetic pathway of CVD. Elevated lipid fractions cause the progression of endothelial damage with the help of inflammatory processes in the vessels. These lipid deposits contribute to stenosis and the occlusion of vessels; hence, they are the actual cause of clinical outcomes [1]. Recent studies have demonstrated that effective treatment of hyperlipidaemia significantly reduces morbidity and mortality due to cardiovascular events [2].

Familial hypercholesterolemia is an autosomal dominant lipid metabolism disorder characterized by high plasma LDL-C (low-density lipoprotein cholesterol) and early atherosclerotic disease. Defaults in genes related to LDL, PCSK9, and apolipoprotein (apo)-B result in an incomplete purge of LDL, leading to an increased concentration of LDL in plasma levels. This disorder has two types of genetic transitions: heterozygous and homozygous. Treatments include statins, bile acid binders, fibric acid derivatives, pharmacological agents (such as omega-3), diet therapy, and lifestyle changes [3].

The human body has a natural antioxidant system that protects cells against oxidative stress. Thiols, the most effective members of this natural antioxidant system, are mercury-binding mercaptans that can be converted into disulfides by oxidation events. Glutathione, cysteine, homocysteine, and N-acetyl cysteine are natural thiols occurring in humans. Thiols have been aggressively researched and have attracted increasing interest recently because of their cancer-mitigating effects. To protect the body from oxidative stress, thiol and disulfide must be in balance [4]. This balance is disrupted in favour of disulfide in many chronic diseases [5]. However, thiol/disulfide homeostasis plays a vital role in the human body.

Recently, important broad human-based studies have demonstrated a strong correlation between oxidative stress and atherosclerosis. Higher levels of oxidized LDL are linked with atherosclerosis. Thus, in impaired antioxidant systems, subendothelial LDL deposition in damaged vessels increases, leading to the formation of foam cells, the initiative factors of atherosclerosis.

It strengthens the notion that proper thiol/disulfide balance is impaired in familial hypercholesterolemic patients, and therefore a disorder in the functioning of the antioxidant system, which increases the oxidation of LDL cholesterol, is the basis for atherosclerotic disease. Increased levels of plasma LDL, as well as the disruption of this antioxidant system by disulfide depletion, initiate the atherosclerotic process in the arterial wall. Interruption of this process may be possible with effective treatment of familial hypercholesterolemia and support of the antioxidant system by improving dynamic thiol/disulfide balance. The aim of this study is to investigate the dynamic thiol/disulfide balance in patients with familial hypercholesterolemia. Our study is the first to examine impaired antioxidant system status in patients with familial hypercholesterolemia.

## 2. Materials and Methods

A total of 51 patients with familial hypercholesterolemia (who were admitted to our endocrinology polyclinic from September 2016 to July 2017) and 81 healthy volunteer subjects were enrolled in our study. The study protocol was approved by the local noninvasive ethics committee and was conducted at the Internal Medicine Department of the Erzurum Regional Education Research Hospital in Erzurum, Turkey. Familial hypercholesterolemia was defined according to the Dutch Lipid Clinic Criteria. Patients suffering from secondary hyperlipidaemia of any aetiology such as diabetes, hypothyroidism, hyperthyroidism, acute or chronic renal insufficiency, adrenal diseases, chronic liver diseases, malignancy, or hyperlipidaemia were excluded. The 51 male and female familial hypercholesterolemia patients did not have a history of smoking, and all patients were from 18 to 79 years of age. The 81 healthy male and female subjects were of similar ages (mean age: 46.84 ± 6.06 years), were nonsmokers, and had body mass index (BMI) values below 30. A comprehensive physical examination was performed, and a detailed family history was obtained from all patients. Height, weight, and waist circumference measurements were recorded for all participants.

Complete blood count, lipid profile, fasting blood glucose, kidney, liver, and thyroid function tests, CRP, and vitamin D levels were evaluated in each patient after at least 12 hours of fasting. Fasting blood glucose, HDL (high-density lipoprotein), LDL, triglyceride, and total cholesterol levels were measured by standard laboratory methods with a Beckman Coulter AU2700 Plus clinical biochemistry analyser (Beckman Coulter, Brea, California, USA). Blood samples were obtained with the oral and written approval of the patients. Plasma samples were centrifuged and then immediately stored in Eppendorf tubes at −80°C in order to investigate thiol/disulfide levels. These blood samples were sent to the Medical Biochemistry Laboratory of Ankara Yıldırım Beyazıt University under appropriate conditions in dry ice for the study of thiol and disulfide levels without denaturation. Samples were analysed on a Roche Cobas 510 analyser (Roche Diagnostics, Indianapolis, USA).

### 2.1. Statistical Analysis

Statistical analysis was performed using the IBM SPSS Statistics version 20 (IBM, Armonk, NY, USA) software program. Data were presented as mean, standard deviation, median, minimum, maximum, percent, and number. The normal distribution of continuous variables was examined by the Shapiro–Wilk test. The independent samples *t*-test was used when normal distribution conditions were provided in the comparison between the two independent groups; the Mann–Whitney *U* test was used when normal distribution conditions were not met. The comparison between the categorical variables was done by the chi-square test and Fisher's exact test. The Pearson correlation test was used if normal distribution conditions were met, and the Spearman correlation test was used if normal distribution conditions were not met when comparing two continuous variables. Receiver operating characteristic (ROC) analysis was used to determine whether the continuous variable could be used in the diagnosis. The relationship between categorical dichotomic dependent variables and independent variables was examined by logistic regression analysis. A *p* value of <0.05 was considered to indicate a statistically significant difference in the 95% confidence interval.

## 3. Results

A total of 132 patients were included in this study, of which 81 were control patients and 51 had a diagnosis of familial hypercholesterolemia. A total of 91.4% of the control group were female (*n*=74), and 8.6% were male (*n*=7); 62.7% of the familial hypercholesterolemia patients were female (*n*=32) and 37.3% were male (*n*=19) (*p* ≤ 0.001).

As can be seen in Table 1, the mean age of the study group was 47 ± 9.17 years. The mean age of the control group was 46.84 ± 6.06 years, and the mean age of the patient group was 47.25 ± 12.64 years. Patient and control groups had similar age distributions (*p*=0.828). None of the participating subjects had a history of smoking. In the patient group, nine patients were on an antihyperlipidaemia diet. BMI medians of the groups were similar (*p*=0.556); the mean BMI in the control group was 29.85 ± 5.17 kg/m^2^, and it was 29.10 ± 5.10 kg/m^2^ in the patient group. In the patient group, 34 were positive for LDL receptor mutation (66.6%), and two (3.9%) were positive for apo-B gene mutation. None of the patients were positive for PCSK9 gene mutation. None of the subjects in the study group were diagnosed with diabetes mellitus; the fasting blood glucose average in the patient group was 92.38 ± 17.65 mg/dl, and it was 113.26 ± 54.82 in the control group (*p*=0.013). The mean LDL cholesterol level was 251.22 ± 51.86 mg/dl in the patient group, whereas the mean LDL cholesterol level was 131.96 ± 44.01 mg/dl in the control group (*p* ≤ 0.001). Only two patients (3.9%) had arcus cornealis and bilateral xanthelasma, and both were positive for LDL receptor mutation. There was no significant difference between the two groups in terms of waist circumference; the waist circumference average was 94.53 ± 9.41 cm in the control group and 91.06 ± 11.84 cm in the patient group (*p*=0.078). Laboratory parameters for the control and patient groups are presented in Table 1.

As can be seen in Table 2, lower levels of native thiol and higher levels of disulfide were independent of BMI, age, and gender in the logistic regression of patients with familial hypercholesterolemia. There was a statistically significant difference between the patient and control groups in terms of the median values of the parameters. It was observed that the results did not change after adjustment of patients and control groups in terms of age, gender, and BMI. There were statistically significant differences between the patient and control groups in terms of the median of the native thiol, total thiol, disulfide, SS/SH%, SS/total SH%, and SH/total SH% parameters. According to these results, we can conclude that these parameters are not affected by age, gender, or BMI.

As can be seen in Table 3, there was a significant negative and poor grade relationship between age and total thiol. There was a significant positive and strong relationship between disulfide and SS/SH%.

**Figure 1:**
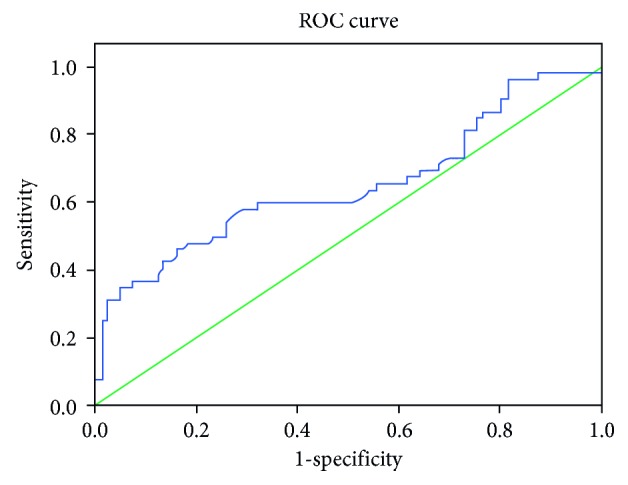
ROC curve analysis for disulfide.

**Figure 2:**
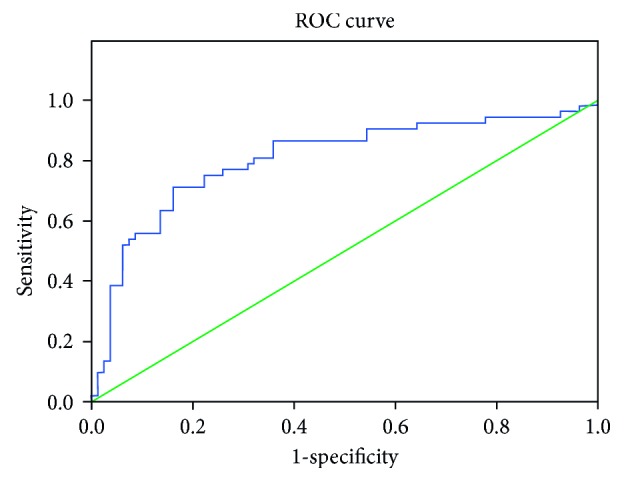
ROC curve analysis for thiol.

A cutoff value of 12.17 for disulfide predicts familial hypercholesterolemia with 57% sensitivity and 73% specificity; 381 for total thiol predicts familial hypercholesterolemia with 76% sensitivity and 70% specificity; and 359 for native thiol predicts familial hypercholesterolemia with 84% sensitivity and 72% specificity (Figures 1 and 2).

## 4. Discussion

This is the first study to evaluate the newly developed dynamic thiol/disulfide balance in patients with familial hypercholesterolemia. Total thiol, native thiol, and native/total thiol levels were lower in the patient group than in the control group. Additionally, disulfide, disulfide/native thiol, and disulfide/total thiol levels were higher in the patient group.

Familial hypercholesterolemia is an autosomal dominant inherited lipid disorder with LDL, apo-B, and PCSK9 mutations that results in persistent LDL elevation and early cardiovascular events. Heterozygous and homozygous genetic forms exist, and the overall frequency in the general population varies from 1/200 to 1/250. Untreated male patients have a 50% higher risk of undergoing cardiovascular events before the age of 50, and female patients have a 30% higher risk of undergoing cardiovascular events before the age of 60 [6]. Mutations in this disease result in a decrease in the hepatic clearance of LDL cholesterol and elevated serum LDL cholesterol levels.

Lower levels of native and total thiol and higher levels of disulfide and SS native thiol were independent of BMI, gender, and age in familial hypercholesterolemia. The change in the balance between thiol and disulfide in favour of disulfide compromises protection from oxidation and redox products. This change causes functional and structural pathologies in most organs and systems. There are natural antioxidant systems in the human body that protect cells from oxidative stress. The dynamic thiol/disulfide balance state plays critical roles in antioxidant defence, detoxification, apoptosis, regulation of enzyme activities, transcription, and cellular signal transduction mechanisms. Thiols are converted to disulfides by oxidation with reactive oxygen radicals. The thiol/disulfide balance is stable in the body. The resulting disulfide bond structures are again reduced to thiol groups so that the thiol/disulfide balance is maintained [7]. Diseases including diabetes mellitus [8], obesity [9], cardiovascular disease [10], hypertension [11], malignancy [12], rheumatic disease, chronic renal failure [13], Alzheimer's disease [14], chronic liver disease [15], cerebral ischemia [16], nasal polyposis [17], preeclampsia [18], uterine myoma [19], cataracts [20], chronic radiation exposure [21], and acute tonsillopharyngitis [22] have been assessed by the same method used in our study, and a close relationship with oxidative stress was detected. A strong association between familial hypercholesterolemia and premature atherosclerotic cardiovascular disease was established in a study conducted with 1,690 patients [1], and, in our study, we found that disulfide formation was elevated in patients with familial hypercholesterolemia. We believe that this is the most likely reason for the incidence of premature atherosclerosis in this group of patients.

Glutathione, cysteine, homocysteine, and N-acetyl cysteine are the most commonly known thiols within the natural human antioxidant system. Many studies show a relationship between oxidative stress and atherosclerosis. Recent studies have shown that oxidative stress and elevated levels of oxidized LDL are very significant factors related to atherosclerosis. In 2016, Kızıltunç et al. demonstrated that plasma native thiol levels were significantly lower and that disulfide levels were significantly higher in patients with coronary artery ectasia [23].

The underlying mechanisms and risk factors of atherosclerosis still remain unclear; however, certain conditions, traits, or habits may raise the chance of developing atherosclerosis. Well-described risk factors including high cholesterol and LDL, low level of HDL in the blood, hypertension, smoking, diabetes mellitus, obesity, sedentary lifestyle, and age can be controlled and atherosclerotic process can be delayed or prevented. LDL oxidation plays a central role in the process of atherosclerosis [24]. Familial hypercholesterolemia is characterized with excessive LDL oxidation, and in this patient population, we observed that total thiol levels were 335.33 ± 78.19 and 410.32 ± 63.95 (*p* ≤ 0.001); native thiol levels were 305.52 ± 71.33 and 389.85 ± 63.31 (*p* ≤ 0.001); and disulfide levels were 18.03 ± 15 and 7810.68 ± 5.87 (*p*=0.004) in the patient and control groups, respectively. Thus, we observed that thiol levels were lower, disulfide levels were elevated, and the thiol/disulfide balance was disrupted in favour of disulfide in patients with familial hypercholesterolemia.

### 4.1. Future Perspectives

Atherosclerotic heart diseases are the most common cause of natural death worldwide. Given that the atherosclerosis process in this disease is triggered by the accumulation of oxidized LDL cholesterol in the vascular wall, reducing oxidized LDL load is a reasonable means to reduce deaths from this disease. A treatment that reduces or prevents LDL oxidation via improvement of dynamic thiol/disulfide balance in favour of thiol could prevent atherosclerotic vascular damage.

### 4.2. Study Limitations

There are two limitations in this study: First, there may be other unknown biochemical and environmental effects that alter the thiol/disulfide balance. Second, the number of patients examined in this study is relatively small because familial hypercholesterolemia is rarer than many other chronic diseases affecting the general population.

## 5. Conclusions

To the best of our knowledge, this is the first study to evaluate oxidative stress with this marker in patients with familial hypercholesterolemia. A new treatment for thiol/disulfide balance may be effective in protecting patients with familial hypercholesterolemia from atherosclerotic heart disease. Further large-scale studies are required to investigate thiol/disulfide balance in patients with familial hypercholesterolemia.

## Figures and Tables

**Table 1:** The mean and p values of parameters of patient and control groups.

Parameter	Patient group	Control group	*p*
	Mean ± SD	Mean ± SD	
Age	47.03 ± 12.67	46.83 ± 6.06	0.903
Gender (female-male)	(74–7)	(32–19)	0.001
Waist circumference	91.06 ± 11.8	94.53 ± 9.41	0.078
BMI	29.10 ± 5.10	29.85 ± 5.16	0.427
N. thiol	305.52 ± 71.33	389.85 ± 63.31	0.001
T. thiol	335.33 ± 78.19	410.32 ± 63.95	0.001
Disulfide	18.03 ± 15.78	10.68 ± 5.87	0.004
%SS/SH	5.76 ± 4.23	2.79 ± 1.37	0.001
%SS/total SH	15.65 ± 76.47	2.63 ± 1.22	0.001
%SH/total SH	91.36 ± 5.16	94.90 ± 1.90	0.001
CRP	4.21 ± 5.20	1.65 ± 2.02	0.001
T. protein (g/dl)	7.34 ± 0.49	21.04 ± 22.45	0.007
Albumin (g/dl)	4.43 ± 0.29	18.73 ± 12.37	0.001
FGL (mg/dl)	92.38 ± 17.65	113.26 ± 54.82	0.013
Creatinine (mg/dl)	0.75 ± 0.14	0.69 ± 0.17	0.343
ALT (U/l)	27.18 ± 16.12	37.58 ± 56.19	0.060
AST (U/l)	23.42 ± 8.12	96.73 ± 70.93	0.002
GGT (U/l)	38.13 ± 29.86	13.18 ± 19.87	0.038
ALP (U/l)	81.26 ± 24.40	26.98 ± 49.54	0.007
Uric acid (mg/dl)	5.62 ± 1.15	4.16 ± 1.67	0.001
T. cholesterol (mg/dl)	339.52 ± 66.54	217.60 ± 74.56	0.004
Triglyceride (mg/dl)	191.74 ± 80.52	168.07 ± 94.58	0.523
HDL (mg/dl)	49.35 ± 9.30	53.78 ± 22.24	0.880
LDL (mg/dl)	251.22 ± 51.86	131.96 ± 44.01	0.001
TSH (uU/ml)	1.70 ± 0.94	1.55 ± 1.22	0.494

ALP: alkaline phosphatase; ALT: alanine aminotransferase; AST: aspartate aminotransferase; BMI: body mass index; CRP: C-reactive protein; FGL: fasting glucose level; HDL: high-density lipoprotein; LDL: low-density lipoprotein; N. thiol: native thiol; T. thiol: total thiol; T. protein: total protein; T. cholesterol: total cholesterol; TSH: thyroid stimulating hormone.

**Table 2:** Regression table.

Parameter	Beta	P value
Age	−0.002	0.949
BMI	−0.020	0.674
Gender	1.988	0.001
Disulfide	−0.277	0.001
Native thiol	1.608	0.001

BMI: body mass index.

**Table 3:** Correlation chart.

Parameter	Native thiol	Total thiol	% SS/SH	% SS/total SH	% SH/total SH
Group					
*p*	−0.529	−0.467	0.465	0.466	−0.427
*r*	0.001	0.001	0.001	0.001	0.001

TC					
*p*	−0.283	−0.239	0.398	0.403	−0.350
*r*	0.004	0.016	0.001	0.001	0.001

TG					
*p*	−0.062	−0.051	0.181	0.181	−0.137
*r*	0.523	0.605	0.062	0.062	0.159

HDL					
*p*	0.015	−0.008	−0.153	−0.152	0.090
*r*	0.880	0.936	0.126	0.127	0.366

LDL					
*p*	−0.352	−0.301	0.420	0.424	−0.374
*r*	0.001	0.002	0.001	0.001	0.001

Gender					
*p*	−0.066	−0.026	0.206	0.212	−0.120
*r*	0.450	0.770	0.018	0.015	0.170

Age					
*p*	−0.296	−0.311	0.068	0.061	−0.114
*r*	0.001	0.001	0.438	0.488	0.193

BMI					
*p*	−0.123	−0.149	−0.012	−0.012	0.001
*r*	0.167	0.094	0.896	0.891	0.987

CRP					
*p*	−0.354	−0.350	0.046	0.044	−0.039
*r*	0.001	0.001	0.681	0.694	0.731

WC					
*p*	0.086	0.047	−0.325	−0.327	0.315
*r*	0.351	0.610	0.001	0.001	0.001

FGL					
*p*	0.036	0.022	−0.010	−0.017	0.003
*r*	0.704	0.814	0.917	0.861	0.978

Crea					
*p*	−0.089	−0.069	0.211	0.217	−0.127
*r*	0.343	0.463	0.022	0.019	0.171

ALT					
*p*	0.060	0.060	0.188	0.198	−0.179
*r*	0.538	0.538	0.053	0.041	0.065

AST					
*p*	0.291	0.264	−0.285	−0.284	0.264
*r*	0.002	0.006	0.003	0.003	0.006

GGT					
*p*	−0.216	−0.209	0.345	0.351	−0.282
*r*	0.038	0.044	0.001	0.001	0.006

ALP					
*p*	−0.271	−0.240	0.220	0.222	−0.192
*r*	0.007	0.017	0.030	0.028	0.058

ALT: alanine aminotransferase; AST: aspartate aminotransferase; ALP: alkaline phosphatase; BMI: body mass index; Crea: creatinine; CRP: C-reactive protein; GGT: gama glutamiltransferase; HDL: high-density lipoprotein; LDL: low-density lipoprotein; T. protein: total protein; TC: total cholesterol; TG: triglyceride.

## Data Availability

Some data are included within the article, and no other data are being made available.
